# Shame-proneness in attempted suicide patients

**DOI:** 10.1186/1471-244X-12-50

**Published:** 2012-05-25

**Authors:** Maria Wiklander, Mats Samuelsson, Jussi Jokinen, Åsa Nilsonne, Alexander Wilczek, Gunnar Rylander, Marie Åsberg

**Affiliations:** 1 Department of Clinical Neuroscience, Karolinska Institutet, Stockholm, Sweden; 2 Department of Clinical Sciences Danderyd Hospital, Karolinska Institutet, Stockholm, Sweden; 3 Department of Neurobiology, Care Sciences and Society, Karolinska Institutet, Stockholm, Sweden

**Keywords:** Attempted suicide, Shame, Borderline personality disorder, Affective disorders

## Abstract

**Background:**

It has been suggested that shame may be an important feature in suicidal behaviors. The disposition to react with shame, “shame-proneness”, has previously not been investigated in groups of attempted suicide patients. We examined shame-proneness in two groups of attempted suicide patients, one group of non-suicidal patients and one group of healthy controls. We hypothesized that the attempted suicide patients would be more shame-prone than non-suicidal patients and healthy controls.

**Methods:**

The Test of Self-Conscious Affect (TOSCA), which is the most used measure of shame-proneness, was completed by attempted suicide patients (n = 175: 105 women and 3 men with borderline personality disorder [BPD], 45 women and 22 men without BPD), non-suicidal psychiatric patients (n = 162), and healthy controls (n = 161). The participants were convenience samples, with patients from three clinical research projects and healthy controls from a fourth research project. The relationship between shame-proneness and attempted suicide was studied with group comparisons and multiple regressions. Men and women were analyzed separately.

**Results:**

Women were generally more shame-prone than men of the same participant group. Female suicide attempters with BPD were significantly more shame-prone than both female suicide attempters without BPD and female non-suicidal patients and controls. Male suicide attempters without BPD were significantly less shame-prone than non-suicidal male patients. In multiple regressions, shame-proneness was predicted by level of depression and BPD (but not by attempted suicide) in female patients, and level of depression and non-suicidality in male patients.

**Conclusions:**

Contrary to our hypothesis and related previous research, there was no general relationship between shame-proneness and attempted suicide. Shame-proneness was differentially related to attempted suicide in different groups of suicide attempters, with significantly high shame-proneness among female suicide attempters with BPD and a negative relationship between shame-proneness and attempted suicide among male patients. More research on state and trait shame in different groups of suicidal individuals seems clinically relevant.

## Background

Suicide can be understood as an act intended to terminate intolerable psychological pain [1,2]. Shame, which is an aversive and often intense affect, could be a source of such psychological pain and some theorists have argued that shame is a core feature in suicidal behaviors (for a review, see [3]). Yet, the relationship between shame and suicidal behaviors has not been much empirically researched. A few qualitative studies on patient experiences have indicated that shame reactions seem to be common after a suicide attempt [4-7]. In an interview study by our research group [6,7], thirteen out of eighteen attempted suicide patients spontaneously described shame reactions after their suicide attempt or during the hospitalization thereafter (e.g., feeling stupid and hesitating to seek help) [6]. Feelings of shame are typically experienced in situations of failure [8,9]. It is understandable that transient feelings of shame, so-called state shame, could be experienced in relation to the suicide attempt (for having failed to cope with life, for having transgressed the social prohibition against suicide, or for having failed to kill oneself) or as a result of circumstances connected with being a psychiatric patient (cf. [10]). In the present study we investigate whether the previous findings of shame reactions also reflect a stable tendency for attempted suicide patients to react with shame, if they are “shame-prone” as a trait. Since attempted suicide is a risk factor for later death from suicide [11-14], an improved understanding of emotional experiences of attempted suicide patients is clinically important.

Theoretically, shame can be described as an aversive affective state paired with a negative evaluation of the entire self [15-17]. Shame is experienced as a more painful and devastating emotion than guilt, because in guilt the negative self-evaluation is restricted to specific behaviors rather than the whole self. Guilt can be alleviated by apology or reparative actions, but few solutions exist to correct the experience of a “faulty” self, and shame typically leads to a wish to hide or escape [18,19]. In a study of use of mental health services in Sweden, Forsell [20] found feelings of shame to be the most common reason for not seeking help for psychiatric problems. In a study by Tangney and Dearing, shame-proneness in fifth grade predicted suicide attempts in early adulthood [21]. Shame has been related to self-injurious behavior with or without suicide intent, in one study of offender women [22] and one study of women with borderline personality disorder (BPD) [23]. Shame-proneness has also been observed generally in BPD women [24-26] and BPD is connected with high rates of attempted and completed suicide [27-29]. However, it is not known whether the high shame-proneness in BPD women is associated with their suicidality. Furthermore, shame has been related to suicidal ideation [30-32] and different expressions of psychopathology (e.g. [33-37]). In line with previous research on shame and suicide related behaviors, we expected that attempted suicide patients generally would be shame-prone.

The aim of the study was to investigate shame-proneness in attempted suicide patients. We hypothesized that attempted suicide patients would be more shame-prone than non-suicidal psychiatric patients and healthy controls. No a priori hypotheses were formulated about differences in shame between suicide attempters with or without BPD, or between male and female participants.

## Methods

### General design

To investigate the relationship between shame-proneness and attempted suicide, the Test of Self-Conscious Affect (TOSCA) [38] was administered to suicidal and non-suicidal participants in four research projects at Karolinska Institutet. Individuals who had attempted suicide up to six months before inclusion in the projects (n = 175) were compared with psychiatric patients (n = 162) and healthy controls (n = 161) with no history of attempted suicide and no current suicide ideation. The TOSCA was distributed together with other questionnaires as part of the research protocols for the respective studies. The investigation of shame-proneness was approved by the Karolinska Institutet Research Ethics Committee, as part of the research protocols for the four studies.

### Definitions of suicidality

The attempted suicide patients included in this study were individuals who had committed an intentional self-destructive act, that he or she thought might be lethal. Superficial self-injuries of low lethality and without intent to die (e.g. cuts, burns, head-banging) were not included in this definition. The non-suicidal comparison groups were psychiatric patients and healthy controls with no previous suicide attempts and no current suicide ideation, as assessed by structured interviews and a score below two on the “Zest for life” item [range 0–3] on the self-rating version of the Montgomery Åsberg Depression Rating Scale (MADRS-S) [39]. All suicidality assessments were performed by experienced physicians.

### Participants

#### Patients

**Figure 1:**
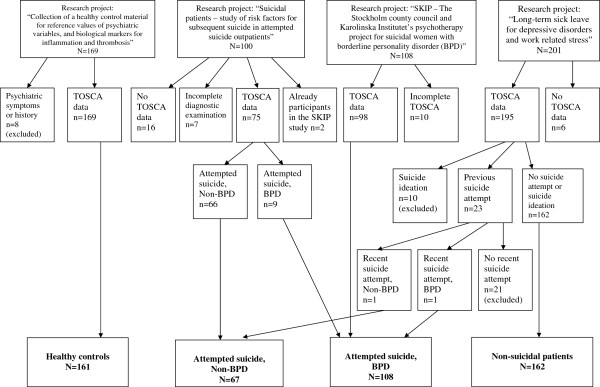
Flowchart of the inclusion process to the present study.

The patients were outpatient participants from two clinical research projects with attempted suicide patients, and one clinical research project with mainly non-suicidal patients. These research projects were investigating: 1) psychotherapy for chronically suicidal women with borderline personality disorder, 2) biological and psychological aspects of attempted suicide, and 3) long-term sick leave for depressive disorders and work-related stress. All patients were diagnosed by experienced psychiatrists, using structured interviews: SCID-I [40] for axis I and DIP-I [41,42] for axis II, according to the DSM-IV [43]. Due to exclusion criteria in the research projects, there were no patients with severe forms of psychosis, anorexia nervosa, substance dependence or melancholia in the present study. Patients from the research projects who met our inclusion criteria of *either* a recent suicide attempt (within six months before inclusion in the projects) *or* no previous suicide attempt and no current suicide ideation, were included in this study. Seventy patients were excluded. Thirty-nine patients were excluded because of missing data (missing or incomplete TOSCA data [n = 32], incomplete diagnostic examination [n = 7]). Thirty-one patients did not fit our inclusion criteria (a history of attempted suicide but no recent suicide attempt [n = 21], no previous suicide attempt but current suicide ideation [n = 10]). Due to the high proportion of female attempted suicide BPD patients in the study, we dichotomized the attempted suicide group into BPD and non-BPD patients and analyzed men and women separately. There were no non-suicidal participants with BPD in the study. The inclusion of study participants are described in Figure 1.

The attempted suicide methods used by the attempted suicide patients were self-poisoning by drugs (n = 86), wrist cutting (n = 9), other cuts (n = 4), hanging/strangulation (n = 5), jumping from a height (n = 1), combined methods (n = 2), and not specified (n = 1) for BPD suicide attempters, and self-poisoning by drugs (n = 45), wrist cutting (n = 6), other cuts (n = 2), attempted drowning (n = 1), jumping from a height (n = 2), jumping in front of moving object (n = 2), crashing of motor vehicle (n = 4), combined methods (n = 4), and other method (n = 1) for non-BPD suicide attempters. Due to inclusion criteria in the research projects, most (97%) BPD patients were attempted suicide repeaters, compared to 33% of the non-BPD suicide attempters.

#### Healthy controls

The healthy controls were a randomized sample, drawn by Statistics Sweden, of Swedish speaking adults participating in a research project collecting data from healthy controls. The healthy controls were assessed for mental health in a screening telephone interview by a trained nurse and an ensuing structured interview (MINI [44] for Axis I, and SCID-II [45] for Axis II) by a trained physician who excluded anyone with a history of psychiatric illness or previous or current suicide attempts or ideation.

### The Test of Self-Conscious Affect (TOSCA)

The Test of Self-Conscious Affect, TOSCA [38], is a widely used scenario-based instrument, assessing proneness to shame, guilt, externalization, detachment, and pride. The pride subscale is divided into pride in oneself (Alpha Pride) and pride in one’s behavior (Beta Pride). The fifteen scenarios depict common situations from social life (e.g., missing an appointment with a friend) and the response alternatives reflect affective, cognitive and behavioral expressions of the variables investigated. The TOSCA was translated into Swedish, using a bilingual committee and a translation back-translation procedure [46]. The Swedish version was back-translated into English and approved by professor June Price Tangney, who is the constructor of the instrument (personal communication, July 19, 1999). The TOSCA has since been revised [47], but only the first version of TOSCA was available at the time of our initial data collection. Although some of the TOSCA subscales have shown low reliability [48], which is common for scenario-based instruments [21], descriptive statistics for all TOSCA subscales are presented to permit comparisons with other studies using the TOSCA.

### Statistical analysis

All statistics were computed with SPSS Statistics 20 [49], or JMP 9.0 [50]. Participants with more than one missing value on any TOSCA subscale were excluded. Internal consistency was calculated with Cronbach’s α. Three attempted suicide men with BPD were excluded from the group comparisons due to small number. Gender differences in shame-proneness within groups, were computed with two-tailed *t*-tests. Gender x group interaction was computed with two-ways ANOVA. Differences between groups were computed with one-way ANOVAs for men and women separately. Post hoc comparisons of group differences were performed with the Tukey-Kramer HSD test. Correlations between predictors of shame-proneness were calculated with Pearson’s *r*. The relationship between shame-proneness and attempted suicide within the patient sample was examined with simultaneous multiple regressions in men and women separately, controlling for age, level of depression, borderline personality disorder, and substance use disorder.

## Results

### Characteristics of the study participants

Participants in the study are presented in Table 1. There were differences between groups regarding group size, gender composition and age. Therefore, men and women were analyzed separately, and age was controlled for in the multiple regressions investigating shame-proneness among psychiatric patients. Level of education differed between groups, but was not related to shame-proneness among psychiatric patients and the variable was therefore not included in the multiple regression analysis.

**Table 1:** Participant groups: basic characteristics and psychiatric disorders (N = 498)

		Attempted Suicide, BPD	Attempted Suicide, Non-BPD	Non-Suicidal Patients	Healthy Controls
N		108	67	162	161
Gender	Female n (*%*)	105 (*97*)	45 (*67*)	117 (*72*)	102 (*63*)
	Male n (*%*)	3 (*3*)	22 (*33*)	45 (*28*)	59 (*37*)
Age	Mean (SD)	29.9 (8.0)	34.8 (12.4)	45.3 (8.9)	44.6 (7.7)
	Range	19-50	18-62	24-60	25-55
Education					
≤9 years	n (*%*)	14 (*13*)	8 (*12*)	24 (*15*)	8 *(5)*
>9-12 years		67 (*62)*	36 (*54*)	75 (*46*)	52 *(32)*
Higher education		27 (*25*)	23 (*34*)	63 (*39*)	101 *(63)*
MADRS-S	Mean (SD)	13.2 (5.6) ¹	10.5 (5.7) ²	7.2 (4.1)	2.3 (1.7)
	Range (0–30)	0-24.5	0-24. 5	0-18.5	0-7.5
Depressive Disorders	n (*%*)	87 (*81*)	50 (*75*)	140 (*86*)	0 *(0)*
Anxiety Disorders	n (*%*)	87 (*81*)	29 (*43*)	27 (*17*)	0 *(0)*
Substance Use Disorders	n (*%*)	21 (*19*)	10 (*15*)	4 (*2*)	0 *(0)*
Eating Disorders	n (*%*)	26 (*24*)	4 (*6*)	3 (*2*)	0 *(0)*
Personality Disorders	n (*%*)	108 (*100*)	11 (*16*)³	20 (*12*)³	0 *(0)*

¹ n = 107.

² n = 66.

³ none of whom had borderline personality disorder.

**Table 2:** TOSCA subscales’ means, standard deviations, and reliability, by group and gender (N = 498)

	N	TOSCA Shame Mean (SD)	TOSCA Guilt Mean (SD)	TOSCA Externalization Mean (SD)	TOSCA Detachment Mean (SD)	TOSCA Alpha Pride Mean (SD)	TOSCA Beta Pride Mean (SD)
Number of items		15	15	15	10	5	5
Range		15-75	15-75	15-75	10-50	5-25	5-25
Reliability		0.80	0.66	0.65	0.68	0.49	0.47
Attempted Suicide BPD	108						
*-*women	105	50.59(8.88)	60.34(7.10)	40.06(7.94)	26.42(6.72)	18.20(3.66)	18.26(3.58)
-men	3	47.67(11.02)	57.00(4.36)	44.00(7.81)	25.67(4.73)	18.67(2.89)	19.00(4.58)
Attempted Suicide Non-BPD	67						
*-*women	45	44.60(9.18)	59.38(5.61)	38.11(6.77)	26.16(7.04)	18.31(3.46)	18.38(3.48)
-men	22	33.18(8.72)	51.32(7.83)	36.36(5.88)	31.66(4.45)	17.09(3.42)	17.23(3.21)
Non-Suicidal Patients	162						
*-*women	117	41.85(8.96)	59.03(5.78)	38.63(7.37)	29.47(6.17)	18.56(3.39)	18.44(3.49)
-men	45	38.33(8.67)	54.47(7.21)	37.42(5.98)	29.93(6.56)	17.07(3.23)	17.51(2.96)
Healthy Controls	161						
*-*women	102	40.93(8.66)	57.38(6.69)	34.54(7.15)	29.02(5.68)	19.26(2.92)	18.82(2.99)
-men	59	37.17(7.33)	52.76(5.91)	33.07(7.44)	29.86(5.86)	18.41(2.65)	18.24(2.50)

### TOSCA

**Figure 2:**
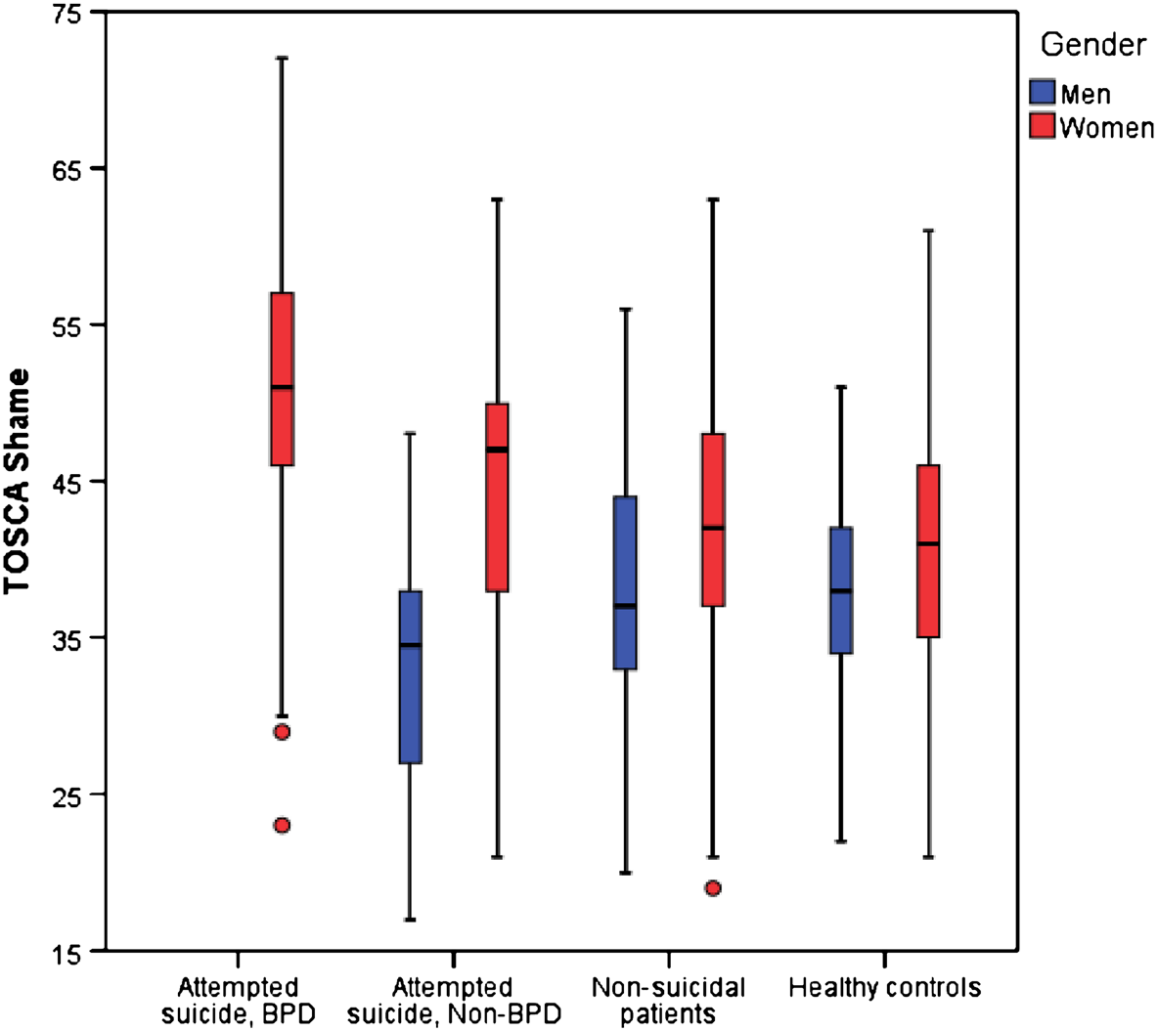
**Distribution of TOSCA Shame by group and gender (N = 495).** Boxes are representing medians and quartiles, whiskers and dots are representing range.

Means, standard deviations, and reliability for all TOSCA subscales, are presented in Table 2. Distribution of TOSCA Shame scores in the participant groups are illustrated in Figure 2. The TOSCA Shame scores of three attempted suicide men with BPD are not presented in the figure due to small numbers. The TOSCA Shame subscale was normally distributed and the internal consistency (Cronbach’s α) was 0.80. The reliability of the other subscales was low, but comparable with other studies [48,51].

### Gender and age differences in shame-proneness

Women had higher shame scores than men of the same participant group (Table 2, Figure 2). The gender differences in shame-proneness within groups were significant in attempted suicide non-BPD patients (*t*[65] = 4.860, *p* < 0.001), non-suicidal patients (*t*[160] = 2.261, *p* = 0.025), and healthy controls (t[159] = 2.806, *p* = 0.006). The gender difference within the attempted suicide BPD group was not calculated due to small numbers in the male group. Shame-proneness was influenced by gender (*F*[1,490] = 13.217, *p* < 0.001), group (*F*[3,490] = 5.108, *p* = 0.002) and gender x group interaction (*F*[3,490] = 3.304, *p* = 0.020). There was a negative correlation between shame-proneness and age (*r*[496] = −0.225, *p* < 0.001).

### Differences in shame-proneness between participant groups

Among female participants, a significant difference between groups was found (*F*[3,365] = 25.409, *p* < 0.001) (Table 2, Figure 2). Post hoc analysis with the Tukey-Kramer HSD test showed that attempted suicide BPD women were more shame-prone than each other female subgroup (*p* = 0.001). Attempted suicide non-BPD women were more shame-prone than female healthy controls, but this difference did not reach statistical significance (*p* = 0.098). In the comparison between male groups, the group of three attempted suicide patients with BPD was excluded due to small numbers. Among the three remaining groups of men, a significant group difference was found (*F*[2,123] = 3.075, *p* = 0.0498) (Table 2, Figure 2). A post hoc analysis with the Tukey-Kramer HSD test showed that attempted suicide non-BPD men were less shame-prone than male non-suicidal patients (*p* = 0.041). Furthermore, it was noted that the few attempted suicide men with BPD had high shame scores.

#### The association between shame-proneness and attempted suicide in the psychiatric patients

To further analyze the association between attempted suicide and shame among the psychiatric patients, multiple regression analyses were performed separately for men and women, adjusting for age, depression severity (as measured by MADRS-S), borderline personality disorder, and substance use disorder. Shame-proneness was used as dependent variable in the model. Pearson’s correlations between all variables in the regression model are presented in Table 3. There were no indications of collinearity between the predictors (tolerance levels ≥ 0.390).

**Table 3:** Pearson’s correlations between predictors for shame-proneness in the psychiatric patients (267 women, 70 men)

Variables	1	2	3	4	5
1. Age	−				
2. Attempted suicide		−			
-women	-.570***				
-men	-.538***				
3. MADRS-S			−		
-women¹	-.215***	.469***			
-men²	-.268*	.274*			
4. BPD				−	
-women	-.502***	.711***	.419***		
-men	-.281*	.284*	.313**		
5. Substance use disorder					−
-women	-.061	.278***	.198**	.256***	
-men	-.118	.252*	.241*	.296*	
6. TOSCA Shame					
-women	-.206**	.352***	.338***	.398***	.173**
-men	.022	-.178	.281*	.244*	.042

¹ n = 266, ² n = 69.

*p < .05. **p < .01. ***p < .001.

The regression model for shame-proneness in female patients was significant (Adj R² = 0.183, *F*[5,260] = 12.874, *p* < 0.001). Borderline personality disorder (*p* = 0.001) and depression severity (*p* = 0.003) were statistically significant predictors for shame-proneness in the regression model, whereas attempted suicide was not a predictor (Table 4). The regression model for shame-proneness in male patients was also significant (Adj R² = 0.136, *F*[5,63] = 3.142, *p* = 0.014). Within the model, depression severity (*p* = 0.020) and non-suicidality (*p* = 0.022) were significant predictors of shame-proneness (Table 4).

**Table 4:** Univariate and multivariate regression effects on shame-proneness in psychiatric patients

	Univariate effects						Model					
	Women (n = 267)			Men (n = 70)			Women (n = 266)			Men (n = 69)		
	B	SE B	β	B	SE B	β	B	SE B	β	B	SE B	β
Intercept							39.307	2.822		34.378	5.791	
Age	−0.177	.052	-.206**	.017	.095	.022	.007	.060	.008	-.009	.108	-.011
Attempted suicide	6.939	1.133	.352***	−3.413	2.283	-.178	1.330	1.754	.067	−6.362	2.719	-.328*
MADRS-S¹	.573	.098	.338***	.563	.235	.281*	.319	.108	.189**	.590	0.246	.295*
BPD	7.973	1.127	.398***	11.025	5.324	.244*	5.258	1.632	.262**	11.272	5.677	.249
Substance Use Disorder	5.903	2.068	.173**	1.051	3.052	.042	1.733	2.035	.050	-.498	3.070	-.020
								Adj R² = 0.183			Adj R² = 0.136	
								F = 12.874***			F = 3.142*	

## Discussion

In this study of trait shame in attempted suicide, three findings add to previous knowledge on the connection between shame and suicidal behavior: male and female suicide attempters differed in the disposition to shame, female suicide attempters with BPD were highly shame-prone, and male non-BPD suicide attempters reported relatively low shame-proneness.

These results, with the highest *and* lowest shame levels found in subgroups of suicide attempters, were unexpected. Previous research has reported *increased* shame only, in relation to suicidal behavior [4-6,21,30,32]. In the studies by Lester [32] and by Hastings and coworkers [30], the relationship was demonstrated by correlations between shame and suicide ideation (Beck Depression Inventory, item 9 [52]; Symptom Checklist 90, item 16 [53]), assessed by questionnaires to college students. In contrast, we examined individuals who were psychiatric patients and had actually attempted suicide. A similar difference between populations may explain the discrepancy between our findings and Tangney and Dearing [21], who found that childhood shame-proneness predicted suicide attempts in adolescence or early adulthood. Even though the young participants in their study reported that they had attempted suicide in their late teens, their degree of psychopathology was likely lower than in our subjects, who were recruited to the study after a suicide attempt severe enough to call for a psychiatric emergency consultation, or referred for treatment of chronic suicidal behavior. The finding of relatively low shame-proneness in male suicide attempters in the present study also challenges findings from a previous qualitative study by our research group, where several male suicide attempters expressed feelings of shame or described shame reactions after their suicide attempt [6]. Though both groups were relatively small and more research is needed to draw any definite conclusions, it is still worth reflecting on the possible meaning of these seemingly contradictory results. The interviews in our qualitative study focused on specific experiences in connection with the suicide attempt, and the shame descriptions could thus be conceptualized as state shame in a desperate situation. The TOSCA inventory, on the other hand, captures propensity to shame in less extreme situations. Low shame-ratings on TOSCA could also be due to difficulties in recognition or acknowledgement of shame feelings. Denial or unawareness of shame is well-known in the theoretical shame literature [15,54], but the phenomenon has rarely been described in empirical research [55]. Our previous interview study also included respondents’ reports of non-verbal shame behaviors (e.g., wanting to hide). Therefore, the respondents themselves did not have to conceptualize their experiences as shame. Furthermore, some suicidal men may be genuinely less shame-prone in everyday life, but still experience shame in connection with a suicide attempt. If these men are unfamiliar with shame experiences or tend to repress such feelings, the shame experience of surviving a suicide attempt might be especially painful. It would thus be important to cautiously help these patients endure the aftermath of the suicide attempt by making psychiatric care easily accessible even if the patients do not express their feelings or communicate their need for help. A respectful and non-demanding stance from the psychiatric personnel may reduce the shame experience of the patient [6].

High levels of shame-proneness were seen among the attempted suicide BPD patients. There were only three men with BPD in the study and more research on shame in this population is thus needed. The finding of high shame-proneness in attempted suicide BPD women is consistent with previous research on shame in BPD [24-26]. It has not been clear whether the shame-proneness in this group is related to the high rates of suicidality in BPD. The results of the present study, with less elevated levels of shame-proneness in attempted suicide women other than BPD, suggest that the shame-proneness is related to the BPD psychopathology and not a reflection of high shame-proneness in suicidal individuals in general. This hypothesis has to be investigated in future studies, which also include non-suicidal BPD participants. Being aware of the high shame-proneness in BPD women can be clinically important. These patients often have difficulties in identifying and expressing their emotions. Shame should be considered when patients exhibit behaviors like non-attendance, silence, and anger [56]. However, patients’ emotions should always be assessed, not assumed [57]. The present study also confirmed the previously described connection between shame-proneness and depression [33,34,37,58,59].

This study has several limitations. Firstly, both the attempted suicide patients and the non-suicidal patients were convenience samples from different clinical research projects. This resulted in a high proportion of women with BPD, relatively few attempted suicide men and only three attempted suicide men with BPD. Thus, future research should include larger groups of attempted suicide men and could also benefit from investigating shame-proneness in diagnostically homogeneous groups of attempted suicide patients other than BPD. The non-suicidal patients were all employees on long-term sick leave for depressive disorders and work related stress. These patients represent only a subgroup of psychiatric outpatients, and their levels of shame-proneness might not be representative for other groups of patients. Thus, shame-proneness in other non-suicidal psychiatric comparison groups should be investigated. Secondly, shame-proneness was measured by self-ratings on a scenario-based instrument. The scenarios in TOSCA might not be appropriate for all populations. For example, several of the scenarios in TOSCA depict work-related situations. However, TOSCA has been used in other studies of psychiatric patients (e.g. [35]) and the patients in our study were outpatients with educational and vocational experiences broadly comparable with non-patients (e.g., more than 95% of the patients had some work experience). A more important concern is if some TOSCA response alternatives may be difficult to acknowledge for groups of respondents, e.g. men who attempt suicide may be less aware of feelings of incompetence or smallness, or hesitate to admit such feelings. One article has also argued that the TOSCA instrument might be less appropriate for male respondents in general [60]. Therefore, future research on shame in suicidal individuals should benefit from investigating shame with several different methods. It must also be remembered that TOSCA measures recognized and admitted propensity to shame in everyday life and that shame-proneness is not a measure of the absolute capacity to experience shame.

## Conclusions

This study is, to our best knowledge, the first empirical investigation of trait shame in groups of attempted suicide patients. Our results indicate that high shame-proneness is not typical for all groups of suicide attempters. However, this does not mean that shame is an unimportant phenomenon in attempted suicide. Shame can be important both as cause, trigger and sequel of a suicidal act, but this does not always reflect a general propensity for the individual to react with shame. More research on shame in suicide, investigated with several different methods, seems clinically relevant.

## Competing interests

The authors declare that they have no competing interests.

## Authors’ contributions

MW, MS, and MÅ conceived of, and designed the study. MW performed the data analyses, interpreted data, and wrote the manuscript. MS, JJ, and MÅ contributed to data analysis and interpretation of data. JJ, ÅN, AW, and GR contributed to study design. JJ, ÅN, AW, GR, and MÅ contributed to acquisition of data. MS, JJ, ÅN, AW, and MÅ contributed in the critical drafting and revising of the manuscript for important intellectual content. All authors read and approved the final manuscript.

## Pre-publication history

The pre-publication history for this paper can be accessed here:

http://www.biomedcentral.com/1471-244X/12/50/prepub
